# The Code

**Published:** 1873-04

**Authors:** 


### THE CODE.

Years ago some wise, old philosopher, having an eye to the exigencies of the case, gave utterance to the exclamation,—"O, consistency, thou art a jewel?" Whether the author of this sentence was a learned disciple of Æsculapius, or was an honored member of a county medical society, we have no means of knowing; but certain it is, or would seem to be, that he had some inkling of our present code of medical ethics; and, contrary to its teachings, this author of concise and wise phrases, at once debarred these societies from practising the sentiment quoted, by patenting it, so outraging the code and doing untold violence to the consciences of the learned members of the societies named. That our hypothesis is correct, we argue, from the fact, that the significance of our quotation is entirely lost upon the members of all medical societies of whom we have any knowledge.

That most members of medical societies are inconsistent, will plainly appear when we note that they habitually violate the code of ethics which they have adopted for their guidance, and then scowl and scold, at those professional gentlemen whom they exclude from membership for doing precisely the same thing. For instance, the code by which all "regular" medical societies are governed, prohibits its members from resorting to public advertisements, private cards, or handbills, or in *any manner* allowing their names to appear in the daily papers, in connection with operations, etc.—Now, to be consistent, the Chemung County Medical Society, and the Elmira Academy of Medicine, should at once expel those members whose names are constantly appearing in our daily papers in connection with various accidents, or as reporters of cases *cured* through certain forms of medication. In fact, both these societies should expel themselves *tout ensemble*, cease the publication of their proceedings in the daily papers, or quit their abuse of those physicians who stand outside the pale for alleged violation of the code. What possible interest the laity can have in the last report of the Chemung County Medical Society, as printed in our daily papers, is more than we can conjecture, unless it be that they have learned that a certain doctor reported a case of "ovarian tumor which had disappeared under his treatment," and that still another had treated with "successful results," a case of *hæmatocele.* If it was not the intention of these medical gentlemen to apprise the suffering public of their superior claims to preferment in the treatment of ovarian tumors, or hæmatocele, why did they not select one of the many excellent medical journals in which to print their report? We do not offer an opinion as to the justice of the code in this particular,—do not say whether it is right or wrong,—but, gentlemen of the societies above named, you have adopted the code for your government, and are not slow to abuse professional gentlemen, not members of your society, for the violation of any article of your code; therefore, stick to it yourselves,—*medecin, gueris-toi toi-meme.*

